# Cine-CMR quantified left atrial diameter - a simple index of left atrial remodeling that closely parallels chamber area and stratifies longitudinal atrial arrhythmic risk

**DOI:** 10.1186/1532-429X-16-S1-P225

**Published:** 2014-01-16

**Authors:** Jiwon Kim, Sergey Gurevich, Jonathan D Kochav, Maya M Petashnick, Anika Afroz, Peter M Okin, Agnes S Kim, Richard B Devereux, Jonathan W Weinsaft

**Affiliations:** 1 Medicine, Weill Cornell Medical College, New York, New York, USA; 2 Medicine, Memorial Sloan Kettering Cancer Center, New York, New York, USA

## Background

Cine cardiac magnetic resonance (cine-CMR) provides excellent endocardial delineation, enabling accurate quantification of left atrial (LA) chamber size. Relative utility of cine-CMR linear dimensions and area-based indices for prediction of LA-associated arrhythmias is unknown.

## Methods

The study comprised patients with coronary artery disease (CAD) included in a multimodality registry. Cine-CMR (1.5T) was performed using a standard 2-dimensional steady state free precession (SSFP) pulse sequence (typical TR 3.5 msec, TE 1.6 msec, flip angle 60°, temporal resolution 30-50 msec). Cine-CMR evidenced mitral regurgitation (MR) severity was graded in accordance with established conventions based on size of MR associated inter-voxel dephasing of the regurgitant jet. LA size was measured on cine-CMR at atrial end-diastole using two established methods: [1] linear diameter (measured in 3-chamber long axis orientation), [2] area (planimetered in 4-chamber long axis orientation), with both indices indexed to body surface area. Clinical follow-up was performed via medical record review, with atrial fibrillation (AF) or flutter (AFl) verified based on physician documentation.

## Results

**Figure 1:**
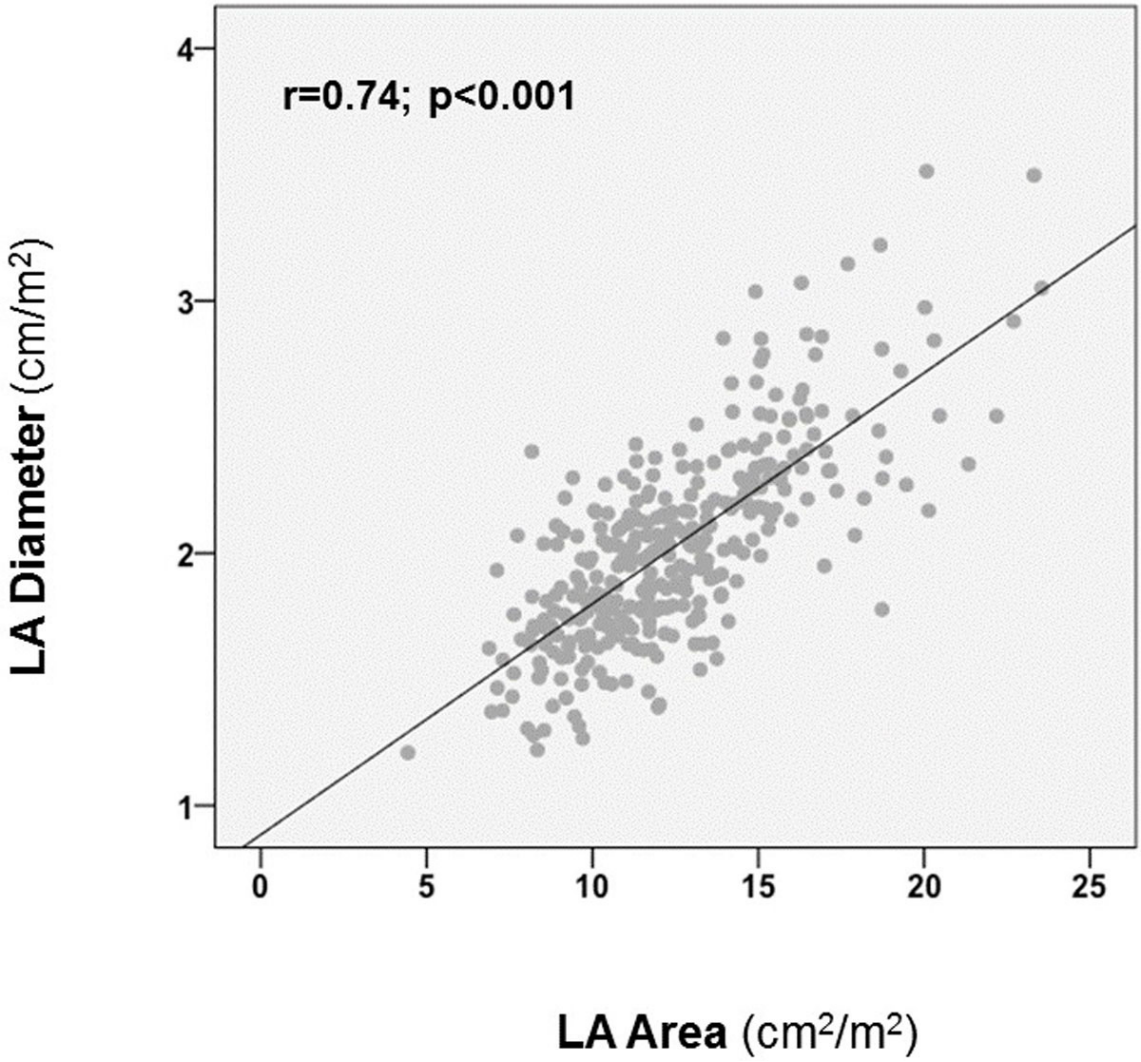
**Scatter plot relating CMR quantified LA diameter and LA area (r = 0.74; p < 0.001)**.

**Figure 2:**
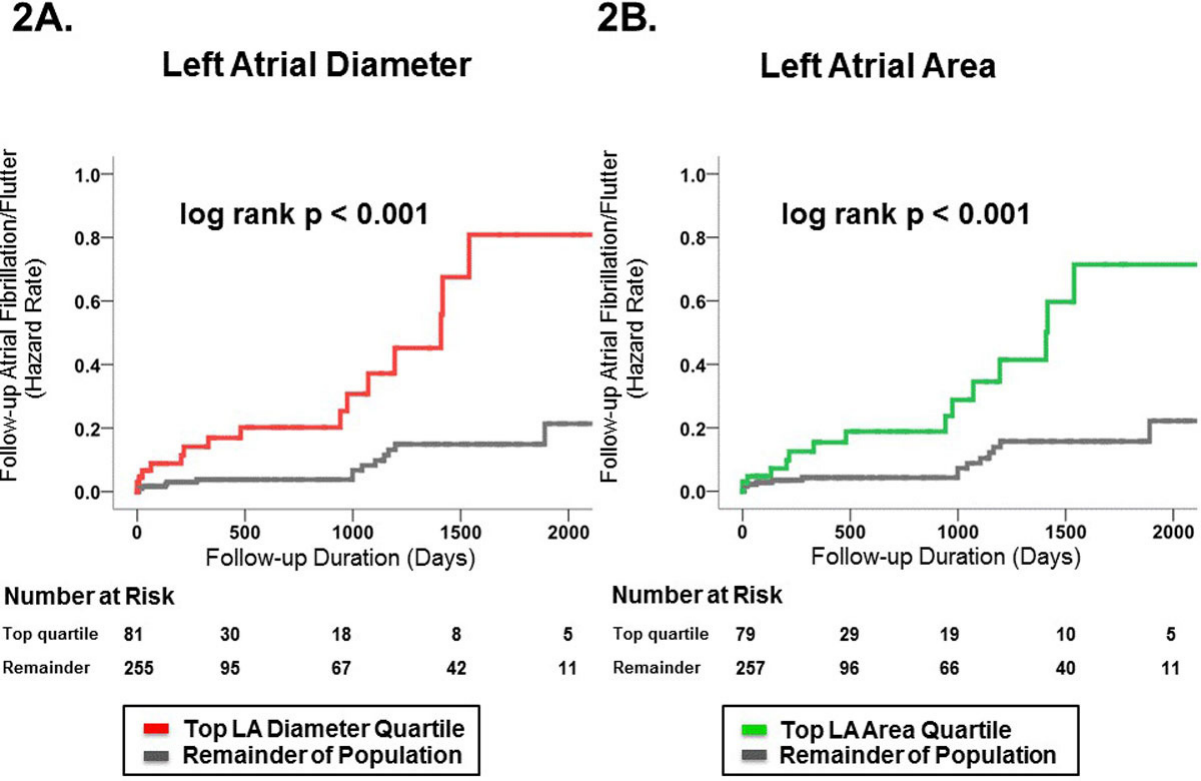
**Kaplan-Meier plots relating baseline LA diameter (2A) and LA area (2B) to follow-up risk for AF/AFl**. Note that both LA diameter and LA area demonstrate increased risk for AF/AFl among patients in the highest quartile of LA remodeling.

336 patients with CAD were studied (60 ± 12 yo, 79% M, 34% DM, 58% HTN); LA diameter (mean 2 ± 0.4 cm/m2) and area (12 ± 3 cm2/m2) yielded similar prevalence of chamber dilation (20% vs. 21%, p = 0.76) assigned using established cine-CMR population-based cutoffs. LA indices correlated highly (r = 0.74, p < 0.001;Figure 1), with similar magnitude of correlation among subgroups at risk for LA remodeling, such as patients with HTN (r = 0.74, p < 0.001) and DM (r = 0.77, p < 0.001). Severe MR was 9-fold more common among patients in the top quartile of LA diameter compared to the remainder of the population (19% vs. 2%, p < 0.001), with similar results when prevalence of severe MR was compared among patients stratified by LA area (17% vs. 3%, p < 0.001). Clinical follow-up (minimum 60 days) was available in 46% of the study population (n = 168). During mean follow-up of 1.5 ± 1.9 years, 31 patients (18%) developed AF/AFl: Cine-CMR quantified LA dimensions stratified arrhythmic risk, with a 5-fold increase in relative risk for AF/Fl among patients in the highest LA diameter quartile (HR 5.1, CI 1.5 - 17.1, p < 0.05), and a 3-fold increase for those in the highest quartile of LA area (HR 3.4, CI 1.1 to 10.6, p < 0.05) compared to the remainder of the population (Figure 2).

## Conclusions

Cine-CMR quantified LA diameter provides a simple measure of atrial remodeling that correlates well with LA area, yielding similar predictive value for stratifying MR as well as longitudinal risk for atrial arrhythmias.

## Funding

National Institutes of Health (K23 HL102249-01).

